# A Unique Case of Gastric Band Tubing Eroding Into the Vaginal Cuff

**DOI:** 10.7759/cureus.68427

**Published:** 2024-09-02

**Authors:** Pranav Balakrishnan, Thomas Adams, Darren B Nease, Semeret T Munie

**Affiliations:** 1 General Surgery, Marshall University Joan C. Edwards School of Medicine, Huntington, USA; 2 Bariatric Surgery, Marshall University Joan C. Edwards School of Medicine, Huntington, USA

**Keywords:** vaginal cuff, robotic bariatric surgery, adjustable gastric band complications, laparoscopic adjustable gastric band, laparoscopic gastric band, bariatric surgery complication

## Abstract

We present here the case of a woman in her 40s with a history of an adjustable gastric band placed a decade ago. After the initial procedure, she had issues with a port-site hernia, mesh placement, and explantation secondary to mesh infection. Her port was removed at the time, with the tubing left in situ with hopes of future salvage. She then presented to her gynecologist with the tubing eroding through her vaginal cuff. This case highlights the importance of having a high index of suspicion in patients with a history of gastric bands given the varying presentation in the event of a complication.

## Introduction

The placement of laparoscopic adjustable gastric bands is falling out of favor [1]. However, bariatric surgeons frequently manage complications from previously placed bands. Gastric band tubing-related complications have variable clinical presentations, making diagnosis extremely challenging. In the case of our patient, her gastric band was initially placed about 10 years prior to her presenting with complications.

This abstract was presented at the American Society for Metabolic and Bariatric Surgery (ASMBS) annual conference on June 28, 2023.

## Case presentation

We present here the case of a woman in her 40s with a history of adjustable gastric band placement at an outside hospital in 2009, who presented to her gynecologist’s office a decade later, with reports of pain during vaginal intercourse. Her partner also reported feeling a hard tubular foreign body in her vagina. On vaginal exam with her gynecologist, there was evidence of white tubing, which broke off during evaluation. She was sent over to the bariatric surgeon as there was suspicion of this being related to her gastric band. 

Of note, six years prior, she had a band port-site hernia which was repaired with mesh. This subsequently became infected, requiring the explantation of the mesh and port. The tubing was left intra-abdominal with a plan to salvage it in the future with the replacement of the subcutaneous port. She was then lost to follow up.

**Figure 1:**
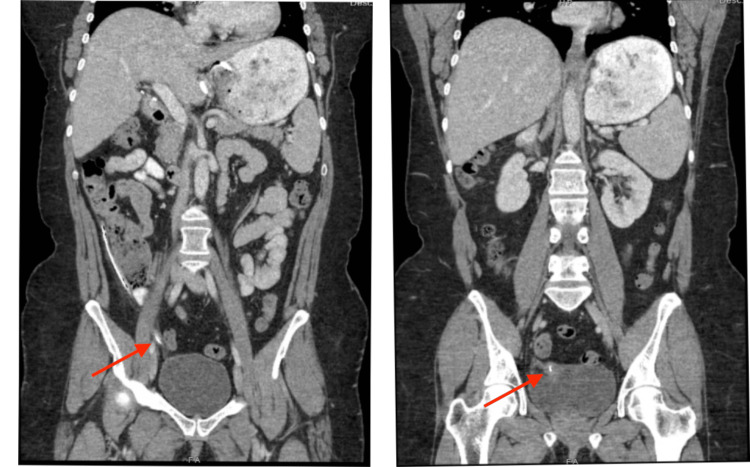
CT imaging revealing the course of the tube in the abdomen (left) and the tubing embedded in the vaginal cuff (right) on sequential coronal sections Red arrows point to the gastric band tubing.

The patient was then evaluated at the bariatric surgery clinic. Imaging showed gastric band tubing embedded in the vaginal cuff (see Figure 1). Esophagogastroduodenoscopy was performed which confirmed that there was no gastric erosion. She was taken to the operating room for a robot-assisted diagnostic laparoscopy, where the catheter and band were removed without incident. The vaginal cuff was evaluated intraoperatively by the gynecologist and a postoperative speculum exam was performed which showed the vaginal cuff to be intact. The patient did well postoperatively and was discharged home and has been doing well on further follow-up visits in the clinic.

To our knowledge, this is the first case report of a patient presenting with gastric band tubing penetrating the vaginal cuff. 

## Discussion

Gastric banding as a modality for bariatric surgery was first used in the early 1980s. This was followed by the introduction of the adjustable gastric band in 1986 [2]. Though the use of adjustable gastric banding has decreased worldwide [1], we continue to see complications stemming from the procedure.

Laparoscopic adjustable gastric banding (LAGB) is a restrictive procedure where an adjustable band is laparoscopically placed around the proximal stomach to create a gastric pouch. This band is connected to a subcutaneous injection port via connecting tubing [3].

Complications associated with LAGB are often divided into major, which usually require abdominal reoperation to resolve, and minor, which are rarely life-threatening and seldom require reoperation. Major complications include band slippage, pouch dilation, and gastric band erosion, while reflux disease, esophagitis, and port and connecting tube problems are considered minor [4]. 

A systematic review by Shen et al. identified the median long-term complication rate associated with LAGB to be 43%, with the reoperation rate being 36.5%. Close to 23% of the patients had their bands removed by the end of the follow-up period. It was found that band slippage and pouch dilation were the most common complications with a median rate of 15.3%. Port/tubing-related complications followed at 11.1%. Other complications identified included band leakage, reflux esophagitis, band erosions, and band infections [5]. Port- or tube-related complications were found to be lower at 6.8% in a five-year prospective study by Steffen et al. [6]. Tube and port complications typically include port dislocation, system leaks, disconnection, and infection [7]. Few report on the penetration of viscera by the free end of the connecting tube in the abdomen.

Complications resulting from LAGB are varied and have an array of presentations. It becomes imperative that all physicians, especially emergency physicians and surgeons, can promptly recognize, diagnose, and treat patients with these complications [3]. A review by Kerpel et al. showed complications identified on computed tomography in 43.1% of 160 patients post LAGB including esophageal dilation, intra-abdominal abscesses, small bowel obstruction, intragastric band erosions, port site and tube course infection, and tube disconnections. Interestingly, less than half of the findings were symptomatic [8].

On reviewing existing reports on connecting tube complications, there were cases of intracolonic penetration of the connecting tube [9,10], migration of the connecting tube into the small bowel [11], and gastric penetration by the connecting tube [12]. The common theme in all these cases was a port-site infection necessitating explantation of the port, followed by the connecting tube being left intra-abdominal with the hope of salvaging the system in the future with replacement of the port at a different location. At the time of salvage surgery or on imaging obtained due to persistent abdominal pain, complications involving the connecting tube were diagnosed. While it is our belief this is the first case report of a patient presenting with gastric band tubing penetrating the vaginal cuff, a similar presentation of foreign body erosion through the vagina has been previously reported due to other intraperitoneal catheters such as in the case of a ventriculoperitoneal shunt [13].

## Conclusions

It is important to have a detailed discussion with patients at the time of port explantation on the risks and benefits of leaving the connecting tube intra-abdominal. Bariatric surgeons must monitor the patient for signs of complications related to the connecting tube if left intra-abdominal, which range from abdominal pain to signs and symptoms of bowel obstruction. Alternatively, the connecting tube and band can be removed in-toto and other bariatric interventions can be offered to the patient.

In the case of our patient, having a detailed discussion regarding LAGB reconnection surgery or revision surgery could have resulted in complete hardware removal years prior and avoided this complication. With the fall in the number of gastric band procedures being performed secondary to complications, and cases like the one presented above, further investigation into the early removal of bands and tubing with complications is warranted.
